# Intercritically Annealed Medium-Manganese Steel: Insights into Microstructural and Microtextural Evolution, Strain Distribution, and Grain Boundary Characteristics

**DOI:** 10.3390/ma17112757

**Published:** 2024-06-05

**Authors:** Sudipta Mohapatra, Kyeong-Cheol Baek, Min-Suk Oh

**Affiliations:** 1 Division of Advanced Materials Engineering, Research Center of Advanced Material Development, Jeonbuk National University, Jeonju 54896, Republic of Korea; dipusudipta57@gmail.com; 2 R&D Department, SAMWOOECO Co., Ltd., Gwangyang 57759, Republic of Korea; kobeon@hanmail.net

**Keywords:** medium-manganese steel, intercritical annealing, microstructure, texture, strain distribution, grain boundary

## Abstract

Aluminum-incorporated medium-manganese steel (MMnS) has potential for lightweight transport applications owing to its impressive mechanical properties. Increasing the austenite volume fraction and making microstructural changes are key to manufacturing MMnS. However, the grain boundary character and strain distribution of intercritically annealed low-density MMnS have not been extensively scrutinized, and the effects of crystallographic texture orientation on tensile properties remain ambiguous. Therefore, in this study, the microstructure, microtexture, strain distribution, and grain boundary characteristics of a hot-rolled medium-Mn steel (Fe–0.2 C–4.3 Al–9.4 Mn (wt%)) were investigated after intercritical annealing (IA) at 750, 800, or 850 °C for 1 h. The results show that the 800 °C annealed sample exhibited the highest austenite volume fraction among the specimens (60%). The duplex microstructure comprised lath-type γ-austenite, fine α-ferrite, and coarse δ-ferrite. As the IA temperature increased, the body-centered cubic phase orientation shifted from <001> to <111>. At higher temperatures, the face-centered cubic phase was oriented in directions ranging from <101> to <111>, and the sums of the fractions of high-angle grain boundaries and coincidence–site–lattice special boundaries were significantly increased. The 800 °C annealed sample with a high austenite content and strong γ-fiber {111}//RD orientation demonstrated a noteworthy tensile strength (1095 MPa) and tensile elongation (30%).

## 1. Introduction

Advanced high-strength steels (AHSSs) have been confirmed to be efficient in developing lightweight structures intended for the locomotive industry [1,2]. Using lightweight metals such as aluminum (Al), magnesium, and titanium offers significant advantages in engineering structures by conserving material and energy. Steels are commonly used in the automotive industry due to their superior mechanical properties, formability, recyclability, and cost-effectiveness [3]. Mass-produced bulk ferrous alloys are often preferred for their cost-effectiveness and suitability for light-weighting purposes. Al alloying is crucial for these alloys because it has a high solubility in iron and can effectively reduce the density of iron. In decreasing the density of steel, thicker gauges can be employed in automobiles, leading to improved fuel efficiency and reduced CO_2_ emissions [4]. The use of AHSSs in automobiles is increasing as technology advances. The cost-effective material is being utilized in various applications, including structure parts, bumper systems, seating, doors, and other safety components. AHSSs are classified into three generations: first, second, and third [5]. First-generation AHSSs encompass dual-phase, complex-phase, and low-Mn martensitic steels (Mn < 3 wt%) that exhibit tensile toughness values (product of strength and elongation, (PSE)) of less than 25 GPa% [6]. Second-generation AHSSs, which are twinning-induced plasticity (TWIP) steels containing austenite with high Mn contents (15–25 wt%), encounter processing and cost issues [7]. Aluminum-added medium-manganese steel (MMnS) is an AHSS that can potentially be used in lightweight transport vehicles [8]. Consequently, significant attention has been devoted to its development, microstructure, and properties [9]. In particular, the Fe–Mn–Al–C system with low carbon contents (≤0.2 wt%), intermediate Mn amounts (5–10 wt%), and moderate Al quantities (1–6 wt%) has drawn research interest owing to its excellent mechanical properties, low density, low material cost, high machinability, and commercial viability [10]. The optimal alloy chemistry, beneficial thermomechanical processing, and effective use of the transformation-induced plasticity (TRIP) mechanism allow MMnS to exhibit remarkable strength and ductility [11,12]. These attractive features are realized by tuning the microstructure, increasing the austenite (γ) volume fraction, and optimizing the mechanical stability of γ [12,13].

At room temperature, carbon stabilizes austenite by influencing its microstructure, solid solution strengthening ability, stacking fault energy (SFE), and tensile properties [14,15]. Although intercritical annealing (IA) causes carbon partitioning, the chemical stability of austenite causes its carbon content to decrease as the IA temperature increases [11,16]. The addition of Mn lowers the martensite start temperature (*M*_s_) by 30–40 °C and the *M*_d30_ temperature (at which 50% of γ is transformed to martensite (α′) at a strain of 30%) by 10 °C, and it lowers the austenite start (*A*_C1_) and finish (*A*_C3_) temperatures [17]. Notably, the volume fraction of γ has been found to increase by ~22% with the addition of ~3–4 wt% Mn [3]. The SFE of austenite in Fe–Mn–Al–C systems initially decreases but increases with increasing Mn concentration, culminating in a parabolic dependence on Mn content [18]. In inhibiting carbide formation, the addition of Al to MMnS lowers density and stabilizes γ [19]. For instance, the addition of 1 wt% Al to steel decreases its density by ~1.5% [19]. Additionally, Al chemically stabilizes ferrite (α) and expands the two-phase (α + γ) domain. Furthermore, fuel economy and carbon emission monitoring are both improved through the use of appropriate amounts of Al [20,21,22]. However, high Al contents (>6%) may result in the formation of kappa carbide, which degrades the mechanical properties of Al-added MMnSs [23,24,25,26]. Nevertheless, by tailoring the composition of Mn–Al–C systems, the mechanical properties can be enhanced, and the deteriorating effect of kappa carbide can be suppressed [27]. For instance, the Fe–8 Mn–6 Al–0.2 C (wt%) steel exhibits a tensile strength of 836 MPa and a tensile elongation (TE) of 32% without any serrations in its stress–strain curve [28]. Moreover, the Fe–10 Mn–1.5 Al–0.14 C (wt%) steel exhibits a strength and ductility of 1045 MPa and 42%, respectively, after undergoing cold rolling and IA treatments [29]. According to Park et al., the deformation-induced martensitic transformation of metastable austenite produces an Fe–8.1 Mn–5.3 Al–0.23 C (wt%) MMnS, which exhibits a tensile strength of 949 MPa and a maximum fracture strain of 54% [30]. Furthermore, the Fe–5.8 Mn–5 Al–0.32 C (wt%) MMnS exhibits a total elongation of 31% and a tensile strength of 950 MPa owing to the beneficial TRIP effect [31].

IA, quenching and tempering, and quenching and partitioning heat treatment schedules are employed for MMnSs [20,32,33,34]. In particular, IA is a notably advantageous processing method as it produces MMnSs with an ultrafine-grained structure that amplifies the TRIP effect [20,32]. Moreover, IA enables the generation of ultrafine lamellar reverted austenite (RA) and intercritical ferrite (IF) microstructures owing to the austenite reversed transformation (ART) of martensite [35]. Notably, intercritically annealed steel undergoes a rapid martensitic transition owing to strain localization in the RA zone. For instance, a PSE value between 25 GPa% and 45 GPa% has been achieved by subjecting Fe–5 Mn–0.2 C (wt%) steel to IA [36]. Moreover, Bai et al. discussed the effectiveness of thermomechanical treatment in modulating the TRIP effect to achieve a high strength–ductility balance [37].

Numerous investigations have demonstrated that high-angle grain boundaries (HAGBs) considerably influence ductility [28,38,39]. Under plastic deformation, HAGBs diverge from the cleavage crack and prevent crack propagation, enhancing ductility. Low coincident site lattice (CSL) boundaries also affect the tensile properties of MMnSs [40]. Interphase boundaries are critical to the plastic deformation of multiphase microstructures because strain partitioning typically occurs in these boundaries with two different microstructural elements on either side. Furthermore, the tensile properties of MMnSs are significantly affected by changes in the texture of face-centered cubic (FCC) austenite and body-centered cubic (BCC) ferrite. Grain size, Mn–C partitioning in austenite, and texture components are certain characteristics that influence the stability of austenite [41,42,43]. Moreover, the strain during deformation, chemical composition, temperature, and prior thermomechanical processing are some aspects that impact the deformation texture [44]. As an important component of rolling direction (RD)//110 fibers, a high intensity of the (113)<110> orientation in the transformation texture evidently improves yield strength [45]. Furthermore, tensile properties are significantly affected by FCC-phase texture components. For example, Barbier et al. found that high textural intensities of the brass component ((110)<1$\bar{1}$2>) lead to improved tensile properties for high-Mn TWIP steel subjected to deformation [46]. However, medium-Mn steel presents unique challenges in this regard. Therefore, the role of the texture components in MMnSs must be studied to reconcile the tensile properties with the TRIP effect.

Additional research is required to identify the most effective combination of the alloying elements—Mn, Al, and C—for realizing MMnSs with superior tensile properties. A potential approach to achieving the intended balance between strength and ductility in Al-added MMnSs is to decrease the number of thermomechanical steps required, which would reduce manufacturing costs. However, few studies have focused on the grain boundary character and strain distribution of low-density MMnSs subjected to IA, particularly those comprising Fe, ≤0.2 wt% C, 3–5 wt% Al, and 8–10 wt% Mn [47,48]. Moreover, the influence of crystallographic texture orientation on the tensile properties of MMnSs has not been extensively investigated. Therefore, the present study aimed at achieving a combination of high strength and ductility (ultimate tensile strength (UTS) > 800 MPa and TE > 25%) by modifying the alloying components (Al, Mn, and C). Thermomechanical processing and IA were currently employed to produce MMnSs containing 0.2 wt% C, 4.3 wt% Al, and 9.4 wt% Mn using melting and casting routes. In this context, the present study was geared toward understanding the microstructural and microtextural evolution at various IA temperatures. Electron backscatter diffraction (EBSD) analysis rooted in an exploration of misorientation angle distributions was performed to provide greater insight into the grain boundary characteristics of annealed specimens. Furthermore, the kernel average misorientation (KAM) method was adopted to examine the strain distribution in the annealed samples. 

## 2. Experimental

### 2.1. Materials and Methods

A steel ingot (162 × 37 × 31 mm^3^) was produced using the conventional melting and casting technique. The chemical composition of the alloy is listed in Table 1. A carbon analyzer was used to detect the C and S contents, whereas an X-ray fluorescence (XRF) device (Bruker D8 Tiger) was employed to measure the concentrations of Fe, Al, Mn, Si, and P.

**Figure 1:**
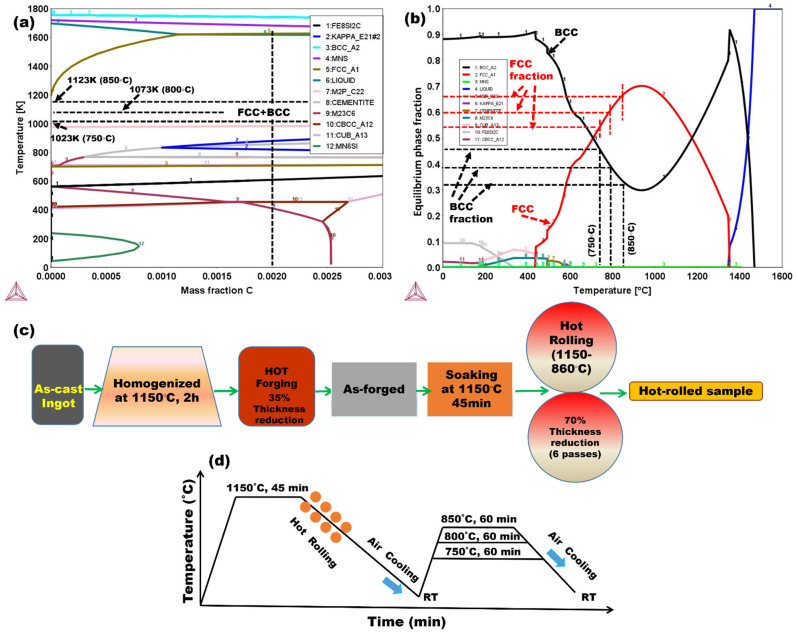
(**a**) Equilibrium phase diagram and (**b**) equilibrium phase fraction diagram of the manufactured steel, constructed using Thermo-Calc software with the TCFE7 database. Schematics of (**c**) the experimental flow diagram and (**d**) the hot-rolling and subsequent heat treatment schedule of the steel.

An equilibrium phase diagram and phase fraction diagram were obtained using Thermo-Calc software with the TCFE7 database (Figure 1). The phase diagram was used to determine the critical temperatures for thermomechanical treatments such as rolling, forging, and annealing. After subjecting the as-cast material to a two-hour homogenization treatment at 1150 °C, the obtained product was forged into a 20 mm thick plate at temperatures ranging from 1150 to 900 °C. A 70% decrease in thickness was achieved using a two-high reverse hot-rolling mill (100-ton load capacity). Prior to being air-cooled to room temperature (25 °C), the forged sample was pre-heated at 1150 °C for 45 min. Subsequently, the specimen was hot-rolled in six passes to a final thickness of 6 mm, maintaining a finish rolling temperature of 860 °C. *A_C_*_1_ and *A_C_*_3_ were estimated to be 660 and >1200 °C, respectively, using the empirical relationships described in Equations (1) and (2) [49]:

(1) $$A_{C1}=723-10.7Mn-16.7Ni+29.1Si+16.9Cr+290As+6.3W$$

(2) $$A_{C3}=910-203\sqrt{C}-30Mn-20Cu-15.3Ni-11Cr-700P+44.7Si+31.5Mo+104V+460Al+13.1W+120As$$

The as-rolled 6 mm thick steel sheets underwent IA for 1 h in a programmed furnace at 750, 800, or 850 °C. These temperatures were selected to be higher than *A_C_*_1_, thereby ensuring annealing in the intercritical domain. The samples annealed to the aforementioned temperatures are denoted as A_750, A_800, and A_850, respectively. The experimental flow diagram, thermomechanical processing, and subsequent heat treatment schedule of the steel are presented in Figure 1c,d.

### 2.2. Microstructural Analysis and Evaluation of Mechanical and Physical Properties

An EBSD system (JIB-4601F, JEOL) and TexSEM Laboratories-Orientation imaging microscopy (TSL-OIM) software were used to construct band contrast phase maps, inverse pole figure (IPF) maps, the microtexture, strain distribution, and grain boundary character distribution (GBCD) of the annealed samples. A cross-sectional area of 200 × 150 µm^2^ was used, and EBSD scanning was performed with a step size of 0.25 µm. The EBSD test samples were prepared via mechanical polishing, followed by electropolishing in a solution containing 90% methanol and 10% perchloric acid [50]. The percentages of low-angle grain boundaries (LAGBs) and HAGBs were classified based on misorientation values of 2°–15° and ≥15°–65°, respectively. Furthermore, X-ray diffraction (XRD; Bruker D8 Advanced) was conducted to confirm the phases present in the annealed samples. The diffraction patterns were obtained using Co-Kα radiation (λ = 1.79 Å) with a step size of 0.02° over a 2θ range of 45°–115°. The volume fraction of austenite in the annealed samples was determined using the direct comparison approach, which involved analyzing the integrated intensities of the diffraction peaks (200)_α_, (211)_α_, (200)_γ_, (220)_γ_, and (311)_γ_ [51]. The following expression (Equation (3)) was used in this regard [52]:

(3) $$V_{\gamma }=\frac{1.4I_{\gamma }}{\left(I_{\alpha }+1.4I_{\gamma }\right)}$$

where *I_γ_* and *I_α_* represent the integrated intensities of the diffraction lines.

Sub-size tensile samples (gauge length, 25 mm; width, 6 mm) were prepared via wire electrical discharge machining in accordance with the ASTM E-8 standard. The annealed specimens underwent strain-controlled room-temperature uniaxial tensile testing using a universal testing machine (INSTRON 8801MTL6258) at a strain rate of 1.33 × 10^−3^ s^−1^. The density of the annealed samples was determined to be 7.35 g/cm^3^ using the Archimedes principle, specifically, the displacement method. This value is 6.48% less than that of pure iron (7.86 g/cm^3^), mostly owing to the incorporation of a high Al content (4.3 wt%).

## 3. Results and Discussion

### 3.1. Microstructural and Microtextural Evolution

**Figure 2:**
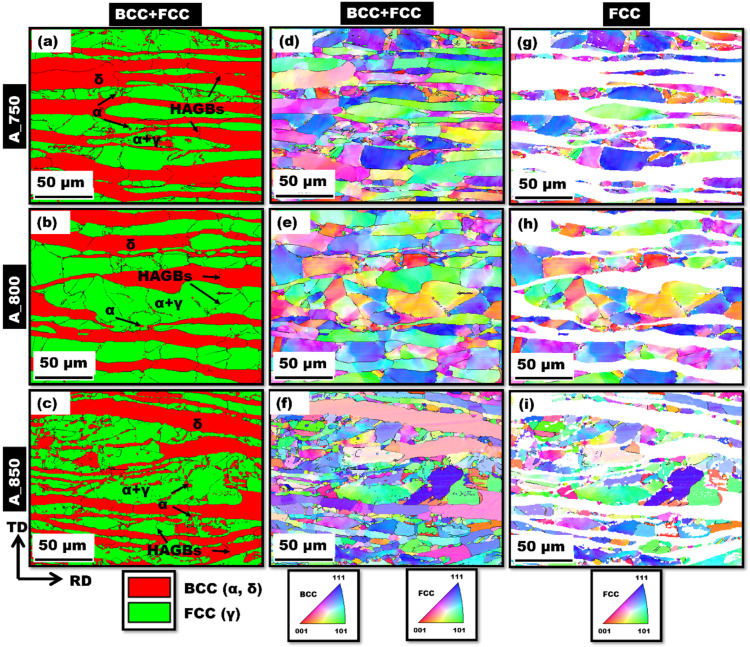
(**a**–**c**) EBSD phase maps and the corresponding (**d**–**f**) IPF maps of annealed samples. (**g**–**i**) IPF maps of FCC phases in annealed samples. (**a**,**d**,**g**) A_750; (**b**,**e**,**h**) A_800; (**c**,**f**,**i**) A_850. (TD: transverse direction, RD: rolling direction, BCC: body-centered cubic, FCC: face-centered cubic, HAGBs: high-angle grain boundaries).

**Figure 3:**
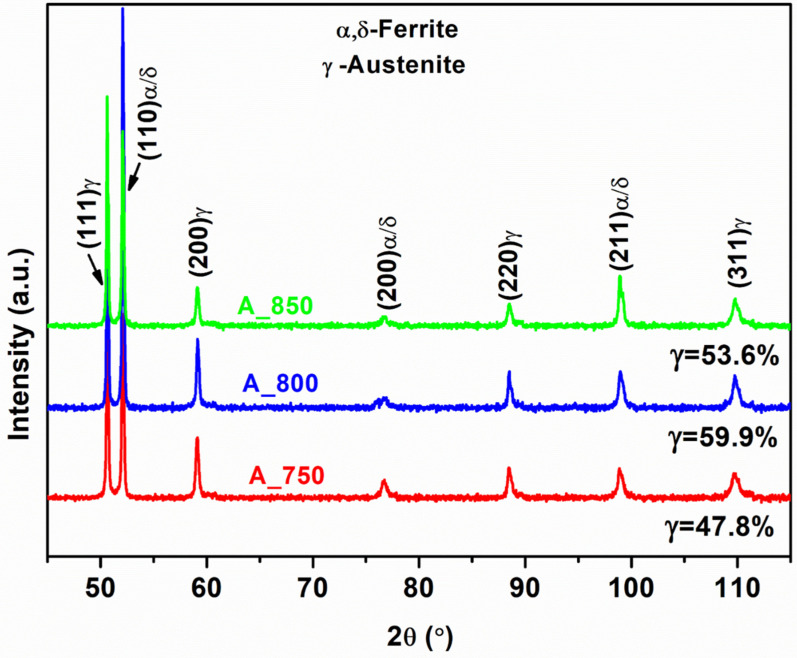
XRD patterns of intercritically annealed samples before the tensile test.

Band contrast EBSD phase maps of A_750, A_800, and A_850 acquired in the transverse direction (TD)–rolling direction (RD) plane are shown in Figure 2a–c, respectively. The annealed samples were found to contain BCC (α- and δ-ferrite) and FCC (γ-austenite) phases (red and green areas, respectively, in Figure 2a–c). The presence of γ-austenite, α-ferrite, and δ-ferrite phases in all the annealed samples was corroborated through XRD analysis (Figure 3), with peaks at (110)_α_, (200)_α_, and (211)_α_ for the ferrite phases and those at (111)_γ_, (200)_γ_, (220)_γ_, and (311)_γ_ for the austenite phase being more prominent in the patterns of the annealed samples. The intercritically annealed steel exhibited a microstructure with alternating layers of primary coarse δ-ferrite and a secondary dual phase, which comprised lamellar γ-austenite islands and fine blocky-shaped α-ferrite. During annealing, α-ferrite and γ-austenite were sequentially formed. According to the related research, the δ-ferrite phase remained unchanged during annealing; however, the γ-austenite and α-ferrite phases were produced via ART [53]. The austenite content increased from 47% to ~60% as the IA temperature increased from 750 to 800 °C. The equilibrium phase fraction diagram (Figure 1b) was considered to establish a correlation between this finding and the observation that a higher austenite fraction is generated at higher IA temperatures. However, the volume percentage of austenite in A_850 (53 vol%) was lower than that of A_800 (~60 vol%). This was presumably due to the reduced availability of Mn and C in austenite at higher IA temperatures (850 °C) and the transformation of a certain amount of austenite (formed during IA at 850 °C) to ferrite during cooling from 850 °C to ambient temperature [54].

**Figure 4:**
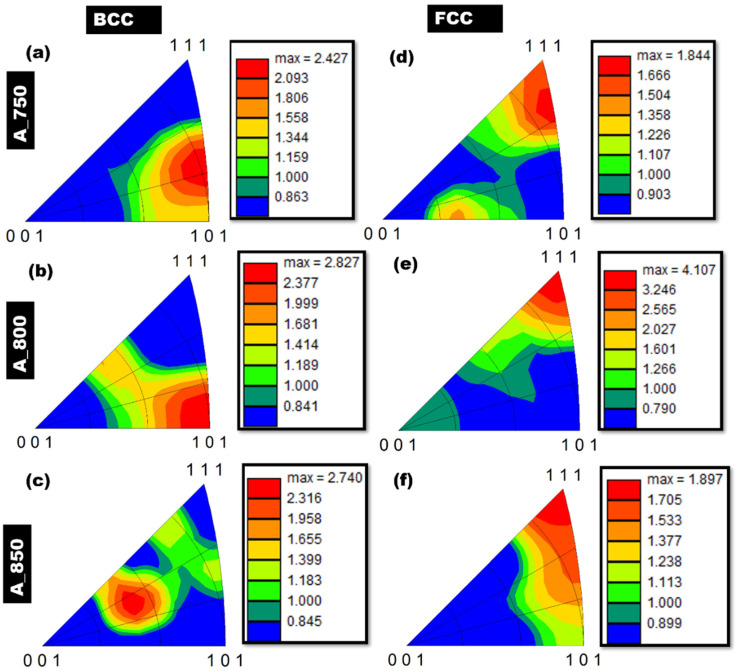
Electron backscatter diffraction inverse pole figure (EBSD IPF) texture maps of (**a**–**c**) BCC and (**d**–**f**) FCC phases in (**a**,**d**) A_750, (**b**,**e**) A_800, and (**c**,**f**) A_850.

The orientations of the phase components were then studied by obtaining IPF maps of A_750, A_800, and A_850 (Figure 2d–f, respectively). A_750 exhibited an orientation of the ferrite phase, namely δ-ferrite, toward the <101> crystallographic direction. Although several grains were oriented between the <101> and <111> directions, the δ phase in A_800 was minimally aligned in the <111> direction. In addition, the δ phase in A_850 was aligned along the <101> direction, whereas other grains were oriented between the <001> and <111> directions. Lath morphologies were exhibited by the γ phase in A_750, with distinct patterns aligned along the <001> to <111> directions (Figure 2g). In contrast, the γ phase in A_800 demonstrated a strong alignment along the crystallographic directions spanning from <101> to <111> (Figure 2h). Moreover, A_850 contained γ with significantly more orientations along the <101> direction; however, it also exhibited an extensive range of crystallographic orientations from <001> to <111> (Figure 2i). These findings were substantiated by IPF texture analysis, which was performed for all the annealed samples with respect to the RD–TD plane (Figure 4a–f).

BCC-phase IPF texture maps of A_750, A_800, and A_850 samples were also acquired, and they are shown in Figure 4a–c, respectively. The IPF texture of the BCC phases (δ and α) in A_750 showed a random texture component with a relative intensity of 2.427R between the <101> and <111> directions (Figure 4a). A higher IA temperature (800 °C) induced a disorderly texture in the BCC phase, particularly in the <001> and <111> directions, with a relative intensity of 1.681R (Figure 4b). Furthermore, the BCC phase of A_800 exhibited a strong α-fiber with a relative intensity of 2.827R along the <101> direction. In the BCC phase of A_850, the IPF texture map showed a random texture component between <001> and <111> directions with a relative intensity of 1.958R (Figure 4c). IPF texture maps of the FCC phase (γ-austenite) in A_750, A_800, and A_850 were also analyzed, and they are illustrated in Figure 4d–f, respectively. For the FCC phase of A_750, the results indicate the formation of γ-fibers in the <111> direction, with a relative intensity of 1.666R (Figure 4d). Moreover, an additional random texture with a relative intensity of 1.504R developed between the <101> and <001> directions. For the FCC phase of A_800, a highly pronounced γ-fiber with a relative intensity of 4.107R was observed in the <111> orientation (Figure 4e). For the FCC phase of A_850, the IPF texture analysis revealed the formation of the γ-fiber in the <111> direction, with a relative intensity of 1.897R (Figure 4f). In summary, an increase in the annealing temperature led to the BCC phase orientation becoming less random and shifting from <001> to <111>. Notably, the FCC phase exhibited a preference between the <001> and <101> directions only in the case of A_750. However, this preference became random as the temperature increased. Moreover, the relative intensity of the γ-fiber in the FCC phase significantly increased from 1.844R to 4.107R when the annealing temperature was increased from 750 to 800 °C.

**Figure 5:**
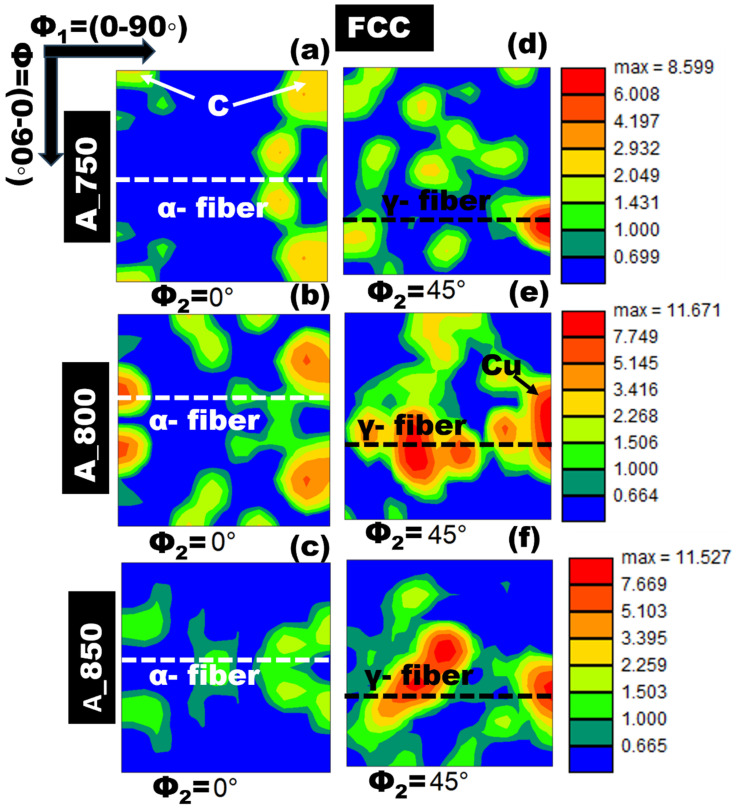
(**a**–**c**) φ_2_ = 0° and (**d**–**f**) φ_2_ = 45° sections of the orientation distribution function (ODF) for FCC-austenite in (**a**,**d**) A_750, (**b**,**e**) A_800, and (**c**,**f**) A_850, with the following important texture components shown in the images: *C*, cube {001} <100>; *α*-fiber {011}//ND; γ-fiber {111}//RD; Cu, copper {112} <111>.

Furthermore, the texture components of the FCC phases in all the annealed samples were assessed using the orientation distribution function (ODF). The ODFs of the FCC-austenite phase in A_750, A_800, and A_850 were plotted in accordance with the Bunge notation for sections with φ_2_ = 0° and 45° (Figure 5a–f, respectively). An increase in the IA temperature led to an increase in the maximum intensity of the texture components. Specifically, the maximum intensity increased from ~8.6R for A_750 to ~11.7R for A_800. Increasing the annealing temperature resulted in the activation of a greater number of potential sites for nucleation [54], which consequently increased the probability of recrystallization. Therefore, grains with a high degree of independent orientation were formed in A_850. Additionally, the {001}<100> cube texture was observed in A_750 (Figure 5a). As the annealing temperature was increased from 750 to 850 °C, grains with random orientation continued to develop while the cube {001}<100> component was annihilated. Moreover, A_800 contained a significant amount of the {112}<111> Cu component (Figure 5e). 

**Figure 6:**
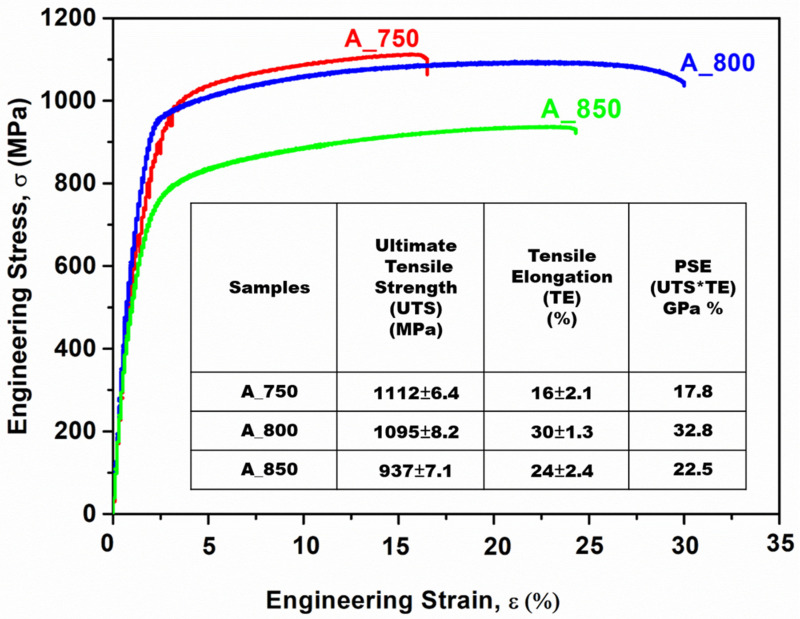
Engineering stress–strain curves of annealed samples.

Furthermore, it showed a noticeable α-fiber orientation with the {011} crystallographic plane parallel to the normal direction (ND). The detrimental cube fibers (ND//<001>) are believed to adversely impact tensile properties and should therefore be minimized [55]. One reason for the inferior combination of tensile strength and elongation of A_750 compared with those of the other two samples is illustrated in Figure 6. The presence of a strong γ-fiber ({111}//RD) orientation can significantly improve mechanical properties [45]. Notably, all the annealed samples in the present study exhibited the γ-fiber orientation ({111}//RD). Among these samples, A_800 showed a higher intensity than that of the others. Overall, the stress–strain analysis (Figure 6) indicated that A_800 exhibited a superior balance between strength and ductility (1095 MPa and 30%, respectively) in contrast to A_750 and A_850.

### 3.2. Strain Distribution in Annealed Samples 

**Figure 7:**
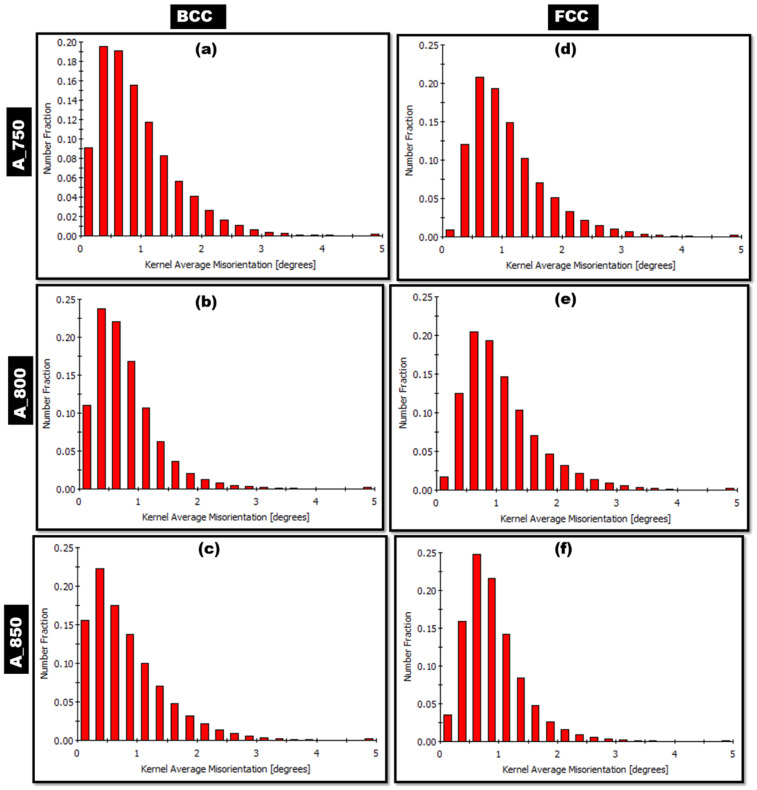
Kernel average misorientation (KAM) distribution graphs of (**a**–**c**) BCC and (**d**–**f**) FCC phases in (**a**,**d**) A_750, (**b**,**e**) A_800, and (**c**,**f**) A_850.

The KAM distribution graphs of the BCC phase in A_750, A_800, and A_850 were subsequently analyzed, and the results are shown in Figure 7a–c, respectively. The fraction of regions with low KAM values (misorientation < 1°) increased and shifted toward 0° as the IA temperature increased to 850 °C. This suggests that the ferrite phase experienced a softening effect, which indicates an annealing-induced relaxation of internal strain and a reduction in dislocation density [56]. Additionally, the KAM distribution graphs of the FCC phase in A_750, A_800, and A_850 are depicted in Figure 7d–f, respectively. The fraction of low-KAM regions increased and shifted toward 0° as the IA temperature increased to 800 °C, similar to the BCC-phase-related findings. Evidently, strain-free austenite was formed as the IA temperature increased. Additionally, the highest peak of the KAM for the FCC phase in A_750 occurred at a higher misorientation angle (>1°) than that of the other two specimens (Figure 7d). The strain relaxation of the FCC phase during annealing was reduced in A_750 owing to two factors. (i) The low percentage of austenite in this sample led to an increase in the amount of the BCC phase. The solute was less likely to be retained by the BCC phase than the FCC phase. The FCC phase in A_750 exhibited a higher solute enrichment than that of the other samples, resulting in a smaller volume for bearing the solute. This enrichment impeded the movement and annihilation of the dislocations, primarily owing to the more pronounced solid solution strengthening [57,58]. (ii) The strain relaxation kinetics were slower because of the lower annealing temperature [59].

### 3.3. Influences of Misorientation Angle and Grain Boundary Character Distribution on Tensile Behavior 

**Figure 8:**
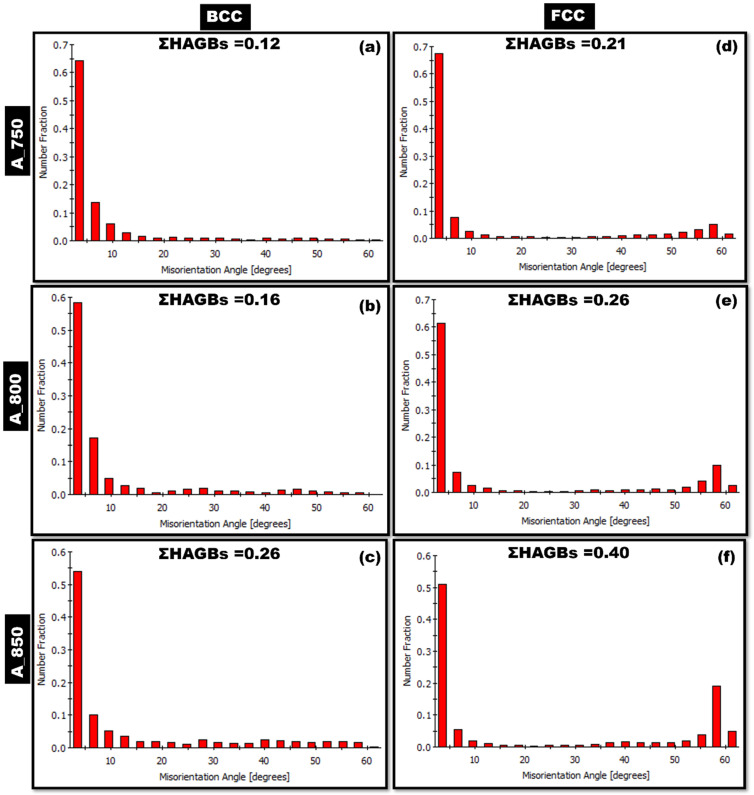
Misorientation angle distribution graphs of (**a**–**c**) BCC and (**d**–**f**) FCC phases in (**a**,**d**) A_750, (**b**,**e**) A_800, and (**c**,**f**) A_850.

**Figure 9:**
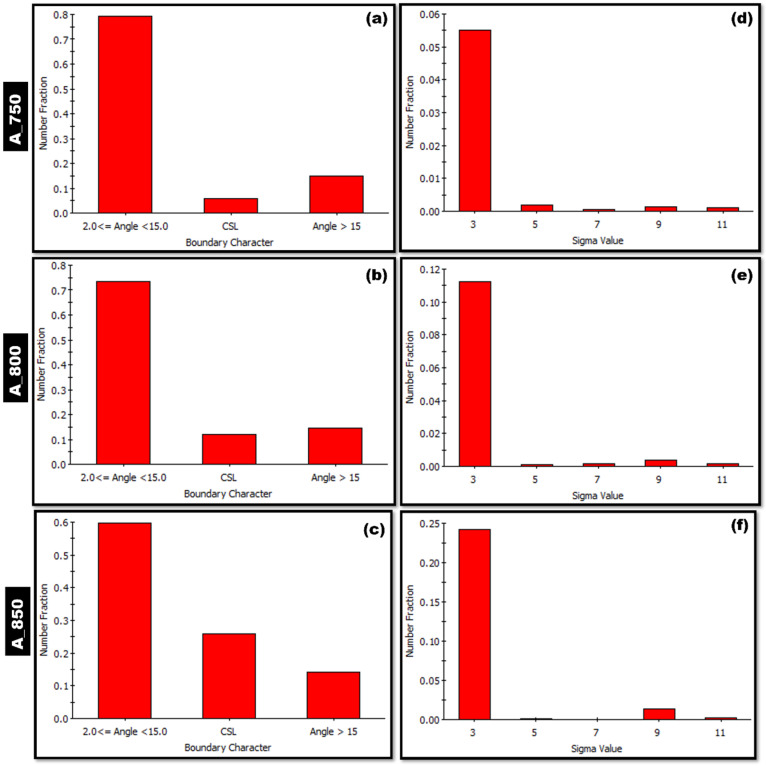
(**a**–**c**) Grain boundary character distribution (GBCD) and (**d**–**f**) coincidence site lattice (CSL) boundary distribution graphs of FCC phase in (**a**,**d**) A_750, (**b**,**e**) A_800, and (**c**,**f**) A_850.

Misorientation angle distribution graphs of A_750, A_800, and A_850 were obtained for the BCC (Figure 8a–c, respectively) and FCC phases (Figure 8d–f, respectively). An increase in the IA temperature from 750 to 850 °C led to an increase in the sum of the fractions of HAGBs (ΣHAGB). In the BCC and FCC phases, ΣHAGB tended to increase from 0.12 to 0.26 and from 0.21 to 0.40, respectively. The observed difference was likely due to the higher fraction of HAGBs (~60°; Figure 8c) and the higher fraction of CSL boundaries in A_850 (Figure 9c). 

The GBCD graphs of the FCC phase in A_750, A_800, and A_850 are shown in Figure 9a–c, respectively, in addition to the corresponding CSL boundary distribution graphs (Figure 9d–f, respectively). The sum of the fractions of grain boundaries with misorientation ≥ 15°, excluding the CSL boundaries—referred to as random HAGBs—increased modestly as the annealing temperature increased from 750 to 850 °C. The sum of the fractions of CSL special boundaries (ΣCSL) increased appreciably with increasing annealing temperatures. The CSL boundaries were calculated considering the specific boundaries Σ3, Σ5, Σ7, Σ9, and Σ11. The CSL boundaries with a misorientation angle of 60° relative to the <111> crystallographic direction are referred to as Σ3, which is characterized by three distinct boundaries [40]. The fraction Σ3 increased as the annealing temperature increased. These Σ3 boundaries are effective in inhibiting crack propagation [60,61]. The misorientation angle axis of 39°/<110> is associated with nine distinct boundaries, which are referred to as Σ9 [61]. In the present study, the fraction of Σ9 boundaries was found to be minimal in A_750, as shown in Figure 9d. Additionally, the contributions of the remaining Σ5, Σ7, and Σ11 CSL boundaries diminished as the annealing temperature increased (Figure 9e,f). 

A_800 exhibited a higher combination of strength and ductility (1095 MPa, 30%, Table 2) than those of A_750 (1112 MPa, 16%) and A_850 (937 MPa, 24%) (Figure 6). This was presumably because A_800 exhibited the highest austenite content (60%) among the specimens (Figure 3). Moreover, studies on HAGBs have indicated that the existence of low CSL boundaries primarily enhances the tensile properties of components by promoting crack resistance [38,61]. The coherent Σ3 CSL boundaries serve as an effective sink for defects and dislocations, dissipating the stored energy in the material, which is generated by the stress field around the crack tip. The presence of these boundaries helps mitigate the impact of the fracture tip by dispersing energy, thus increasing the crack propagation resistance [38,60]. Therefore, A_800 showed a strong strength–ductility relationship owing to its higher ΣHAGB value (FCC phase; 0.26; Figure 8e) than that of A_750 (FCC phase; 0.21; Figure 8d). Additionally, A_800 exhibited a lower ΣCSL value (0.12; Figure 9b) than that of A_850 (0.23; Figure 9c). Notably, A_750 showed inadequate ductility owing to its relatively low austenite volume fraction (47.8%; Figure 3) despite having an ΣCSL value of 0.05 (Figure 9a). Table 2 lists the tensile properties of the present steel in comparison to previously published works. The current steel exhibits an excellent UTS of 1095 MPa and a substantial degree of ductility, surpassing that of some works with comparable compositions.

## 4. Conclusions

Lightweight MMnSs were developed by controlling the IA temperature of Fe–9.4 Mn–4.3 Al–0.2 C (wt%) steel, and modifications in microstructure and microtexture were examined in detail. Furthermore, the synergistic effects of IA temperature on strain distribution and GBCD were examined. The salient conclusions of this study are outlined below:

1. The IA treatment resulted in blocky-type α-ferrite and lath-type austenite within a coarse δ-ferrite matrix. An increase in the annealing temperature from 750 to 850 °C led to increases in the proportions of HAGBs present in the BCC and FCC phases.
2. The δ-ferrite phase in A_750 and the γ-austenite phase in A_850 were oriented along the <101> crystallographic direction. γ-austenite in A_750 showed a preference for the <001> and <101> directions. In contrast, γ-austenite in A_800 was oriented from <101> to <111>.
3. As the annealing temperature was increased from 750 to 850 °C, grain formation became more random, and the undesired cube component ({001}<100>) was eliminated. Moreover, the α-fiber ({011}//ND) and copper components ({112}<111>) were present in A_800. In addition, the annealed samples featured a prominent γ-fiber ({111}//RD) orientation, with A_800 exhibiting the highest intensity.
4. A_750 showed the highest KAM peak intensity among the specimens, with a misorientation angle exceeding 1°. Increasing the IA temperature caused a shift toward 0°, indicating strain-free austenite formation.
5. A_800 exhibited a more pronounced correlation between tensile strength (1095 MPa) and tensile elongation (30%) owing to its higher austenite content and lower ΣCSL value than those of A_850. However, A_750 exhibited insufficient ductility owing to its low austenite volume fraction and low ΣHAGBs fraction.

Collectively, these findings suggest that the lightweight MMnS with 4.3 wt% Al and 9.4 wt% Mn exhibits a remarkable strength–ductility combination. Hence, this steel shows promise as a candidate with low alloying costs and substantial weight reduction capabilities for new automotive applications. Our future research on this steel will include an investigation of the martensitic transformation mechanism that occurs during tensile testing. In addition, we will focus on investigating biaxial and triaxial stress states, which are of greater significance for steel structures used in the automotive sector. 

## Figures and Tables

**Table 1:** Nominal chemical composition of the investigated steel.

Element	Al	Mn	Si	P	C	S	Fe
Concentration (wt%)	4.3	9.42	1.27	0.02	0.2	0.01	Balance

**Table 2:** Comparison of tensile properties of the present work with other works.

Composition (wt%)	UTS (MPa)	TE (%)	PSE (GPa%)
Fe–10Mn–3.19Al–0.2C [18]	793	28	22.20
Fe–10Mn–1.5Al–0.14C [29]	1045	42	43.89
**Fe–9.4Mn–4.3Al-0.2C (present work)**	**1095**	**30**	**32.85**
Fe–8.1Mn–5.3Al–0.23C [30]	949	54	51.24
Fe–8.1Mn–2.85Al–0.2C [27]	1000	18.5	18.50
Fe–8Mn–6Al–0.2C [28]	836	32	26.75
Fe–7.85Mn–3.83Al–0.27C [41]	878	64	56.19
Fe–7.15Mn–3.21Al–0.2C [18]	802	19	15.23
Fe–6.72Mn–3.92Al–0.18C-1.34Si [62]	803	61	48.98
Fe–6.5Mn–3Al–0.2C–0.1V [39]	966	42.6	41.15
Fe–5.8Mn–5Al–0.32C [31]	950	31	29.45
Fe–5.0Mn–0.05C–0.5Si–1.4Ni–0.12V [63]	960	28.5	27.36

## Data Availability

The raw data supporting the conclusions of this article will be made available by the authors on request.

## Nomenclature

AHSS, advanced high-strength steel; MMnS, medium-manganese steel; PSE, product of strength and elongation; TWIP, twinning-induced plasticity; TRIP, transformation-induced plasticity; IA, itercritical annealing; RA, reverted austenite; ART, austenite reversed transformation; HAGBs, high-angle grain boundaries; CSL, coincident site lattice; KAM, kernel average misorientation; IPF, inverse pole figure; GBCD, grain boundary character distribution; ODF, orientation distribution function.
